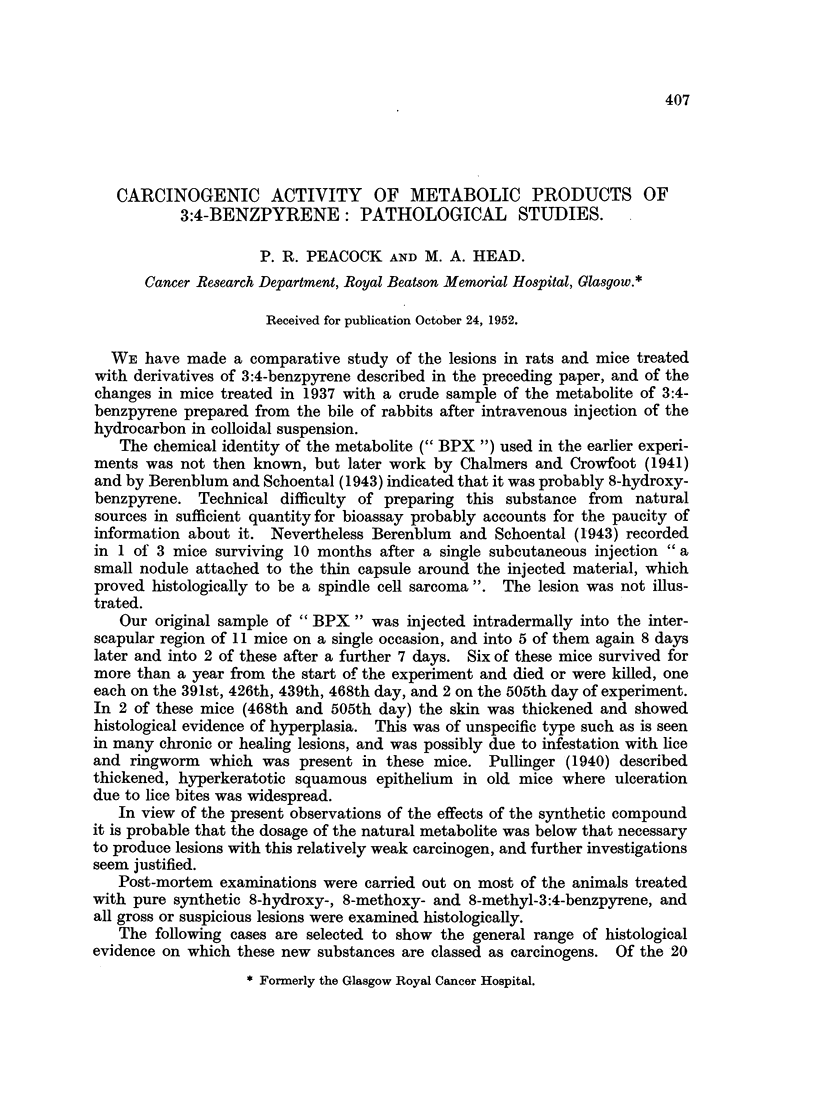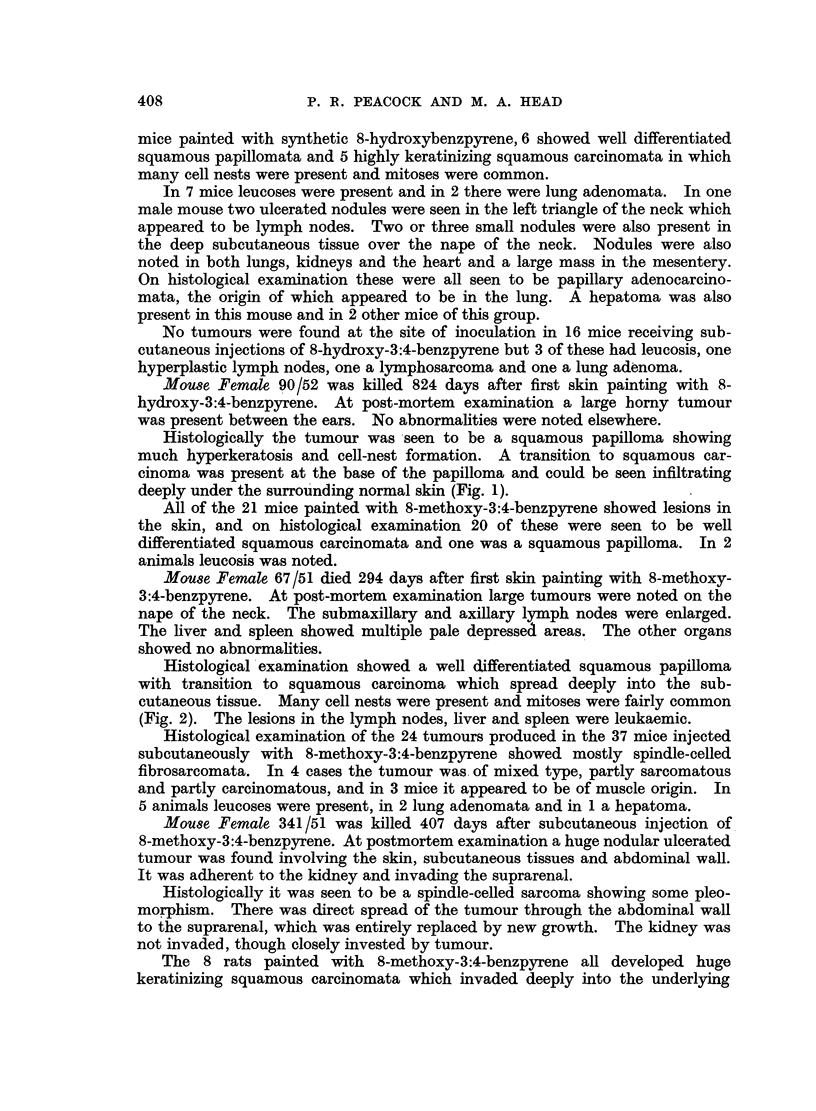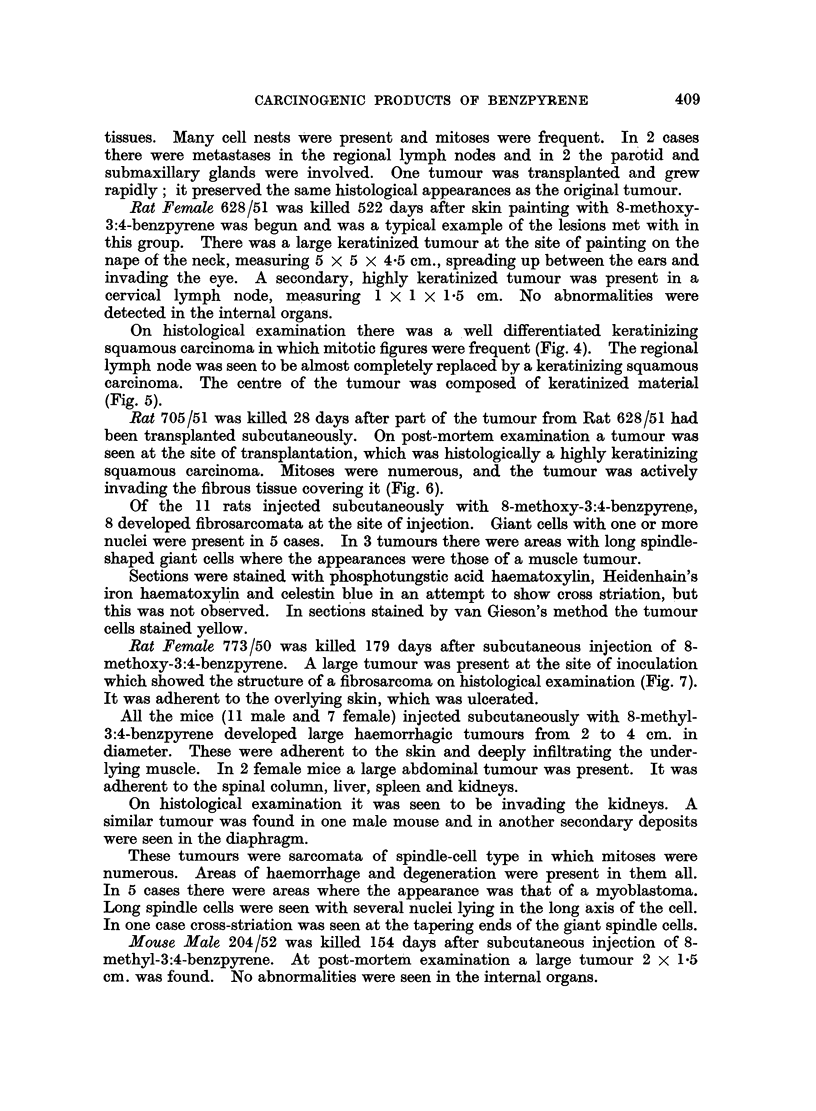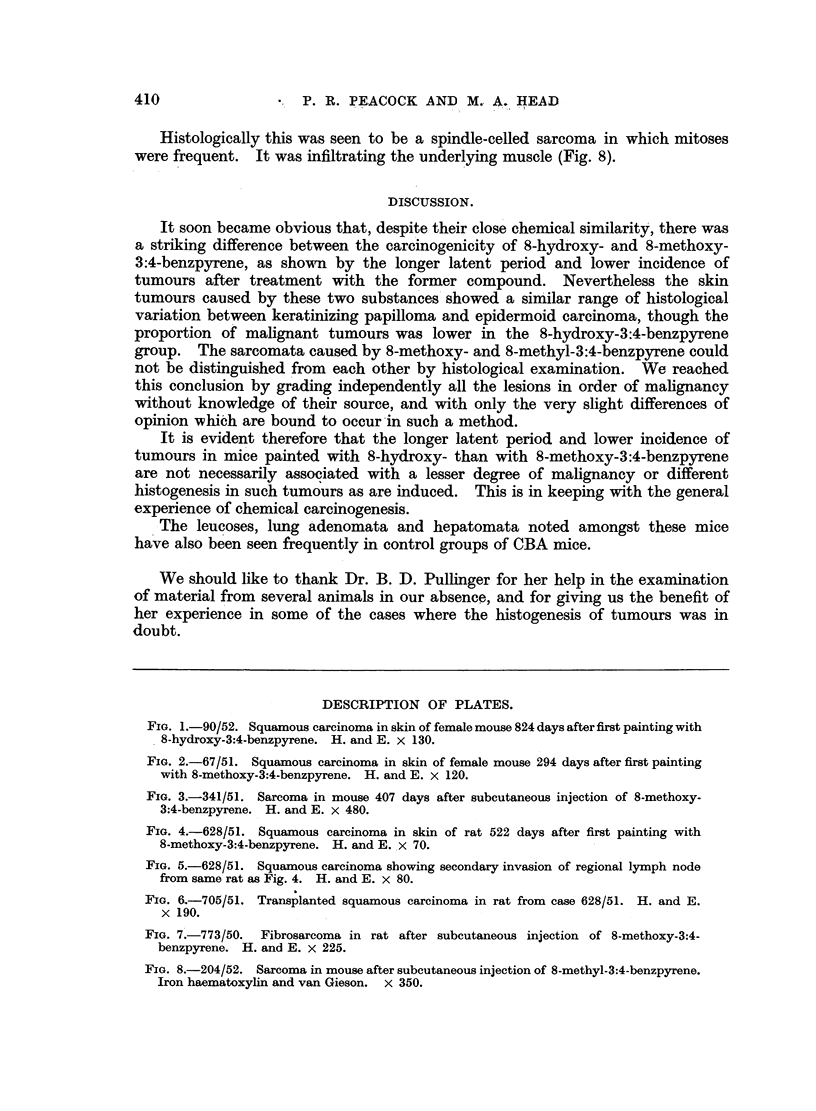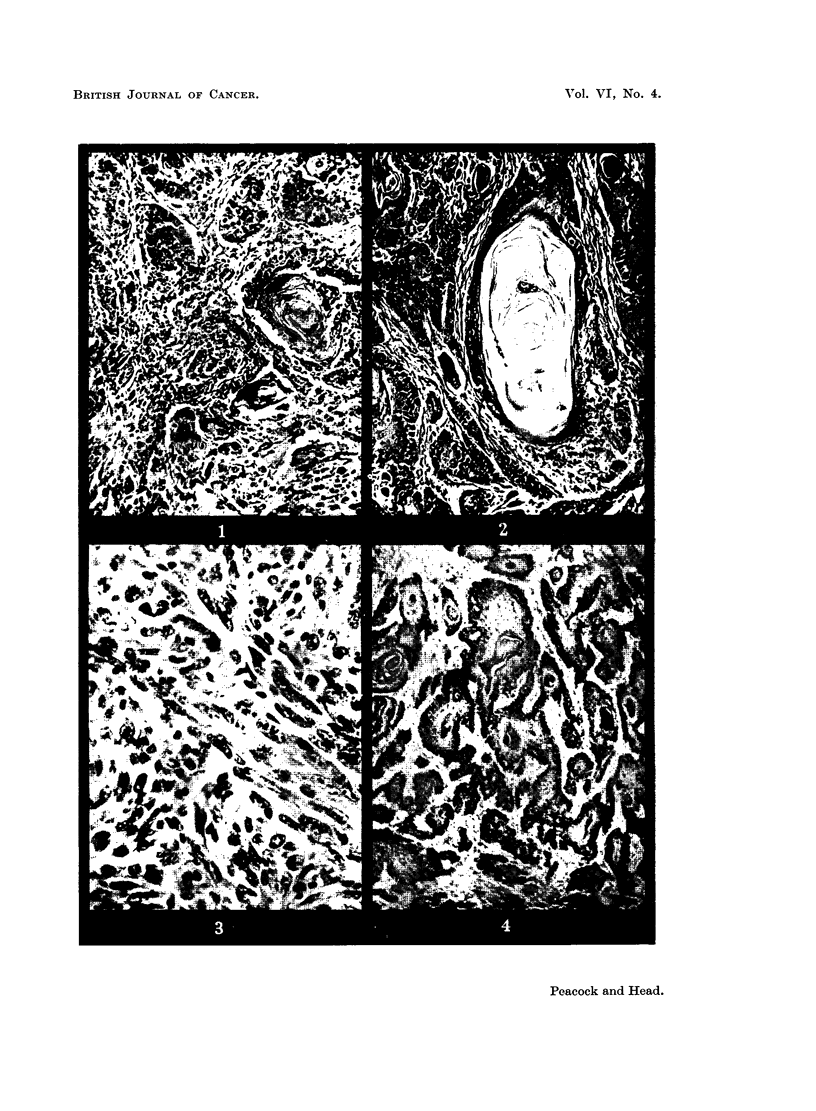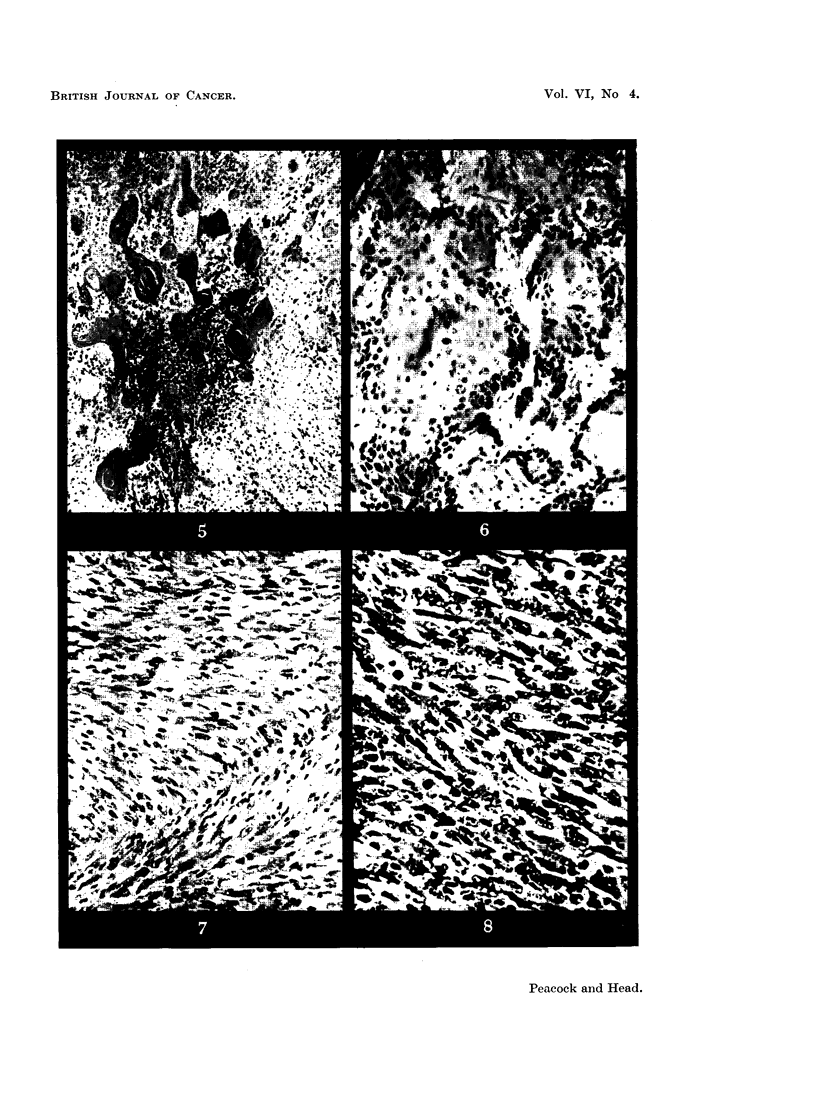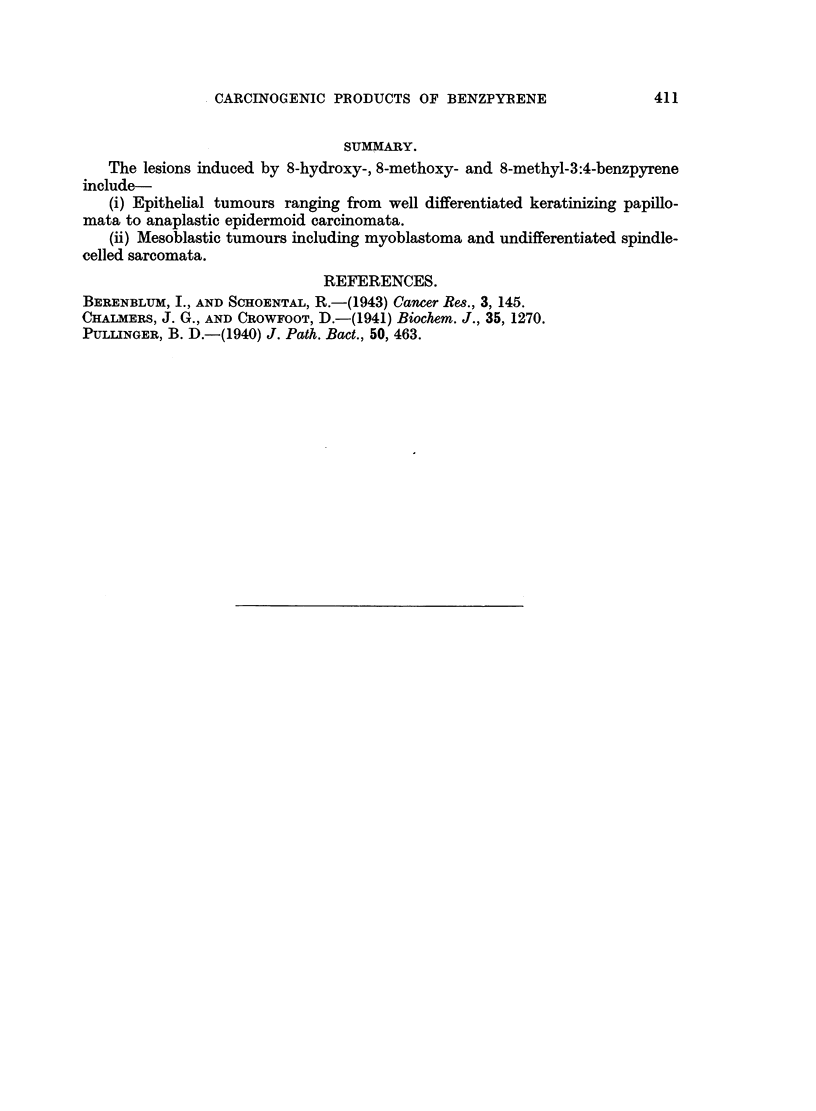# Carcinogenic Activity of Metabolic Products of 3:4-Benzpyrene: Pathological Studies

**DOI:** 10.1038/bjc.1952.46

**Published:** 1952-12

**Authors:** P. R. Peacock, M. A. Head

## Abstract

**Images:**


### CARCINOGENIC ACTIVITY OF METABOLIC PRODUCTS OF 3:4-BENZPYRENE: PATHOLOGICAL STUDIES.

P. R. PEACOCK AND M. A. HEAD.

*Cancer Research Department, Royal Beatson Memorial Hospital, Glasgow.**

Received for publication October 24, 1952.

WE have made a comparative study of the lesions in rats and mice treated with derivatives of 3:4-benzpyrene described in the preceding paper, and of the changes in mice treated in 1937 with a crude sample of the metabolite of 3:4-benzpyrene prepared from the bile of rabbits after intravenous injection of the hydrocarbon in colloidal suspension.

The chemical identity of the metabolite (" BPX ") used in the earlier experiments was not then known, but later work by Chalmers and Crowfoot (1941) and by Berenblum and Schoental (1943) indicated that it was probably 8-hydroxy-benzpyrene. Technical difficulty of preparing this substance from natural sources in sufficient quantity for bioassay probably accounts for the paucity of information about it. Nevertheless Berenblum and Schoental (1943) recorded in 1 of 3 mice surviving 10 months after a single subcutaneous injection " a small nodule attached to the thin capsule around the injected material, which proved histologically to be a spindle cell sarcoma ". The lesion was not illustrated.

Our original sample of " BPX " was injected intradermally into the inter-scapular region of 11 mice on a single occasion, and into 5 of them again 8 days later and into 2 of these after a further 7 days. Six of these mice survived for more than a year from the start of the experiment and died or were killed, one each on the 391st, 426th, 439th, 468th day, and 2 on the 505th day of experiment. In 2 of these mice (468th and 505th day) the skin was thickened and showed histological evidence of hyperplasia. This was of unspecific type such as is seen in many chronic or healing lesions, and was possibly due to infestation with lice and ringworm which was present in these mice. Pullinger (1940) described thickened, hyperkeratotic squamous epithelium in old mice where ulceration due to lice bites was widespread.

In view of the present observations of the effects of the synthetic compound it is probable that the dosage of the natural metabolite was below that necessary to produce lesions with this relatively weak carcinogen, and further investigations seem justified.

Post-mortem examinations were carried out on most of the animals treated with pure synthetic 8-hydroxy-, 8-methoxy- and 8-methyl-3:4-benzpyrene, and all gross or suspicious lesions were examined histologically.

The following cases are selected to show the general range of histological evidence on which these new substances are classed as carcinogens. Of the 20

* Formerly the Glasgow Royal Cancer Hospital.

mice painted with synthetic 8-hydroxybenzpyrene, 6 showed well differentiated squamous papillomata and 5 highly keratinizing squamous carcinomata in which many cell nests were present and mitoses were common.

In 7 mice leucoses were present and in 2 there were lung adenomata. In one male mouse two ulcerated nodules were seen in the left triangle of the neck which appeared to be lymph nodes. Two or three small nodules were also present in the deep subcutaneous tissue over the nape of the neck. Nodules were also noted in both lungs, kidneys and the heart and a large mass in the mesentery. On histological examination these were all seen to be papillary adenocarcinomata, the origin of which appeared to be in the lung. A hepatoma was also present in this mouse and in 2 other mice of this group.

No tumours were found at the site of inoculation in 16 mice receiving subcutaneous injections of 8-hydroxy-3:4-benzpyrene but 3 of these had leucosis, one hyperplastic lymph nodes, one a lymphosarcoma and one a lung adenoma.

*Mouse Female* 90/52 was killed 824 days after first skin painting with 8-hydroxy-3:4-benzpyrene. At post-mortem examination a large horny tumour was present between the ears. No abnormalities were noted elsewhere.

Histologically the tumour was seen to be a squamous papilloma showing much hyperkeratosis and cell-nest formation. A transition to squamous carcinoma was present at the base of the papilloma and could be seen infiltrating deeply under the surrounding normal skin (Fig. 1).

All of the 21 mice painted with 8-methoxy-3:4-benzpyrene showed lesions in the skin, and on histological examination 20 of these were seen to be well differentiated squamous carcinomata and one was a squamous papilloma. In 2 animals leucosis was noted.

*Mouse Female* 67/51 died 294 days after first skin painting with 8-methoxy-3:4-benzpyrene. At post-mortem examination large tumours were noted on the nape of the neck. The submaxillary and axillary lymph nodes were enlarged. The liver and spleen showed multiple pale depressed areas. The other organs showed no abnormalities.

Histological examination showed a well differentiated squamous papilloma with transition to squamous carcinoma which spread deeply into the subcutaneous tissue. Many cell nests were present and mitoses were fairly common (Fig. 2). The lesions in the lymph nodes, liver and spleen were leukaemic.

Histological examination of the 24 tumours produced in the 37 mice injected subcutaneously with 8-methoxy-3:4-benzpyrene showed mostly spindle-celled fibrosarcomata. In 4 cases the tumour was of mixed type, partly sarcomatous and partly carcinomatous, and in 3 mice it appeared to be of muscle origin. In 5 animals leucoses were present, in 2 lung adenomata and in 1 a hepatoma.

*Mouse Female* 341/51 was killed 407 days after subcutaneous injection of 8-methoxy-3:4-benzpyrene. At postmortem examination a huge nodular ulcerated tumour was found involving the skin, subcutaneous tissues and abdominal wall. It was adherent to the kidney and invading the suprarenal.

Histologically it was seen to be a spindle-celled sarcoma showing some pleomorphism. There was direct spread of the tumour through the abdominal wall to the suprarenal, which was entirely replaced by new growth. The kidney was not invaded, though closely invested by tumour.

The 8 rats painted with 8-methoxy-3:4-benzpyrene all developed huge keratinizing squamous carcinomata which invaded deeply into the underlying tissues. Many cell nests were present and mitoses were frequent. In 2 cases there were metastases in the regional lymph nodes and in 2 the parotid and submaxillary glands were involved. One tumour was transplanted and grew rapidly ; it preserved the same histological appearances as the original tumour.

*Rat Female* 628/51 was killed 522 days after skin painting with 8-methoxy-3:4-benzpyrene was begun and was a typical example of the lesions met with in this group. There was a large keratinized tumour at the site of painting on the nape of the neck, measuring 5 × 5 × 4·5 cm., spreading up between the ears and invading the eye. A secondary, highly keratinized tumour was present in a cervical lymph node, measuring 1 × 1 × 1·5 cm. No abnormalities were detected in the internal organs.

On histological examination there was a well differentiated keratinizing squamous carcinoma in which mitotic figures were frequent (Fig. 4). The regional lymph node was seen to be almost completely replaced by a keratinizing squamous carcinoma. The centre of the tumour was composed of keratinized material (Fig. 5).

*Rat* 705/51 was killed 28 days after part of the tumour from Rat 628/51 had been transplanted subcutaneously. On post-mortem examination a tumour was seen at the site of transplantation, which was histologically a highly keratinizing squamous carcinoma. Mitoses were numerous, and the tumour was actively invading the fibrous tissue covering it (Fig. 6).

Of the 11 rats injected subcutaneously with 8-methoxy-3:4-benzpyrene, 8 developed fibrosarcomata at the site of injection. Giant cells with one or more nuclei were present in 5 cases. In 3 tumours there were areas with long spindle-shaped giant cells where the appearances were those of a muscle tumour.

Sections were stained with phosphotungstic acid haematoxylin, Heidenhain's iron haematoxylin and celestin blue in an attempt to show cross striation, but this was not observed. In sections stained by van Gieson's method the tumour cells stained yellow.

*Rat Female* 773/50 was killed 179 days after subcutaneous injection of 8-methoxy-3:4-benzpyrene. A large tumour was present at the site of inoculation which showed the structure of a fibrosarcoma on histological examination (Fig. 7). It was adherent to the overlying skin, which was ulcerated.

All the mice (11 male and 7 female) injected subcutaneously with 8-methyl-3:4-benzpyrene developed large haemorrhagic tumours from 2 to 4 cm. in diameter. These were adherent to the skin and deeply infiltrating the underlying muscle. In 2 female mice a large abdominal tumour was present. It was adherent to the spinal column, liver, spleen and kidneys.

On histological examination it was seen to be invading the kidneys. A similar tumour was found in one male mouse and in another secondary deposits were seen in the diaphragm.

These tumours were sarcomata of spindle-cell type in which mitoses were numerous. Areas of haemorrhage and degeneration were present in them all. In 5 cases there were areas where the appearance was that of a myoblastoma. Long spindle cells were seen with several nuclei lying in the long axis of the cell. In one case cross-striation was seen at the tapering ends of the giant spindle cells.

*Mouse Male* 204/52 was killed 154 days after subcutaneous injection of 8-methyl-3:4-benzpyrene. At post-mortem examination a large tumour 2 × 1·5 cm. was found. No abnormalities were seen in the internal organs.

Histologically this was seen to be a spindle-celled sarcoma in which mitoses were frequent. It was infiltrating the underlying muscle (Fig. 8).

### DISCUSSION.

It soon became obvious that, despite their close chemical similarity, there was a striking difference between the carcinogenicity of 8-hydroxy- and 8-methoxy-3:4-benzpyrene, as shown by the longer latent period and lower incidence of tumours after treatment with the former compound. Nevertheless the skin tumours caused by these two substances showed a similar range of histological variation between keratinizing papilloma and epidermoid carcinoma, though the proportion of malignant tumours was lower in the 8-hydroxy-3:4-benzpyrene group. The sarcomata caused by 8-methoxy- and 8-methyl-3:4-benzpyrene could not be distinguished from each other by histological examination. We reached this conclusion by grading independently all the lesions in order of malignancy without knowledge of their source, and with only the very slight differences of opinion which are bound to occur in such a method.

It is evident therefore that the longer latent period and lower incidence of tumours in mice painted with 8-hydroxy- than with 8-methoxy-3:4-benzpyrene are not necessarily associated with a lesser degree of malignancy or different histogenesis in such tumours as are induced. This is in keeping with the general experience of chemical carcinogenesis.

The leucoses, lung adenomata and hepatomata noted amongst these mice have also been seen frequently in control groups of CBA mice.

We should like to thank Dr. B. D. Pullinger for her help in the examination of material from several animals in our absence, and for giving us the benefit of her experience in some of the cases where the histogenesis of tumours was in doubt.

---

### DESCRIPTION OF PLATES.

FIG. 1.—90/52. Squamous carcinoma in skin of female mouse 824 days after first painting with 8-hydroxy-3:4-benzpyrene. H. and E. × 130.

FIG. 2.—67/51. Squamous carcinoma in skin of female mouse 294 days after first painting with 8-methoxy-3:4-benzpyrene. H. and E. × 120.

FIG. 3.—341/51. Sarcoma in mouse 407 days after subcutaneous injection of 8-methoxy-3:4-benzpyrene. H. and E. × 480.

FIG. 4.—628/51. Squamous carcinoma in skin of rat 522 days after first painting with 8-methoxy-3:4-benzpyrene. H. and E. × 70.

FIG. 5.—628/51. Squamous carcinoma showing secondary invasion of regional lymph node from same rat as Fig. 4. H. and E. × 80.

FIG. 6.—705/51. Transplanted squamous carcinoma in rat from case 628/51. H. and E. × 190.

FIG. 7.—773/50. Fibrosarcoma in rat after subcutaneous injection of 8-methoxy-3:4-benzpyrene. H. and E. × 225.

FIG. 8.—204/52. Sarcoma in mouse after subcutaneous injection of 8-methyl-3:4-benzpyrene. Iron haematoxylin and van Gieson. × 350.

1
2
3
4

5

6

7

8

Peacock and Head.

SUMMARY.

The lesions induced by 8-hydroxy-, 8-methoxy- and 8-methyl-3:4-benzpyrene include—

(i) Epithelial tumours ranging from well differentiated keratinizing papillomata to anaplastic epidermoid carcinomata.

(ii) Mesoblastic tumours including myoblastoma and undifferentiated spindle-celled sarcomata.

### REFERENCES.

Berenblum, I., and Schoental, R.—(1943) *Cancer Res.*, **3**, 145.
Chalmers, J. G., and Crowfoot, D.—(1941) *Biochem. J.*, **35**, 1270.
Pullinger, B. D.—(1940) *J. Path. Bact.*, **50**, 463.